# Prevalence and predictors of pediatric high blood pressure: an Egyptian school-based survey

**DOI:** 10.1186/s12889-026-27360-x

**Published:** 2026-04-23

**Authors:** Ehab Elrewany, Hassan Farag Mohamed Farag, Sherif Omar Osman, Samah Batouta Saleh, Doaa Tawfik Mohamed

**Affiliations:** 1 Department of Tropical Health, High Institute of Public Health, Alexandria University, Alexandria, Egypt; 2 Department of Sports Training and Movement Sciences, Faculty of Sports Sciences for Girls, Alexandria University, Alexandria, Egypt; 3 Department of Nutrition, High Institute of Public Health, Alexandria University, Alexandria, Egypt

**Keywords:** Children, Hypertension, Elevated blood pressure, School-based survey

## Abstract

**Background:**

Childhood hypertension (HTN) is a substantial public health problem due to its rising trend and strong correlation with adulthood HTN with its long-term sequelae. It has notable unmodifiable and modifiable risk factors. The current study aimed to estimate the prevalence of high blood pressure (BP); (elevated BP and HTN), among preparatory schoolchildren in El-Beheira Governorate, Egypt and to identify correlates associated with high BP.

**Methods:**

Using a multistage random sampling, from El Beheira governorate 15 districts, Damanhur was chosen where 4 preparatory schools (two for boys + two for girls) and 856 students aged 11–14 years were recruited for a cross-sectional study. A pre-designed structured interview questionnaire having socio-demographic information; habitual and family history data; besides anthropometric and three BP measurements were obtained for each student. BP was classified according to the new American normative BP in healthy children and adolescents.

**Results:**

Students boys and girls were equally distributed (50%), with an average age of 13.3 ± 0.66 years. Among the students, 27% were obese/overweight and 40% didn’t exercise regularly. The frequency of elevated BP and HTN was 9.1% and 6.9%, respectively. High BP predominated with girls (crude odds ratio (COR) = 2.811, 95% confidence interval (CI); 1.891–4.178), physical inactivity (≤ once weekly: COR = 30.366, 95% CI; 15.424–59.785, & 2–3 times weekly: COR = 19.364, 95% CI; 10.253–36.571), high risk diet (COR = 4.302, 95% CI; 2.944–6.289), added table salt (COR = 13.374, 95% CI; 8.037–22.257), family history of HTN (COR = 2.003, 95% CI; 1.330–3.018), overweight (COR = 3.718, 95% CI; 2.243–6.161), obesity (COR = 8.444, 95% CI; 5.322–13.399), and abdominal obesity (COR = 4.016, CI; 2.744–5.877), (*p* < 0.05). The multivariate analysis revealed that physically activity ≤ once weekly (adjusted odds ratio (AOR) = 15.679, 95% CI; 6.936–35.443) & 2–3 times weekly physical activity (AOR = 10.738, 95% CI; 5.247–21.973), usage of added table salt (AOR = 5.745, 95% CI; 3.108–10.617), being overweight (AOR = 2.735, 95% CI; 1.298–5.765) and obese (AOR = 7.463, 95% CI; 3.414–16.314) were the predictors for having high BP level among the students, (*p* < 0.05).

**Conclusions:**

Screening of schoolchildren has explicitly unveiled the high frequency of high BP (elevated BP and HTN) among preparatory schoolchildren tipped the scales in favor of the importance of applying preventive strategies that emphasize screening and early detection of childhood elevated BP and HTN and conducting longitudinal prospective studies to establish causality of the identified associated factors.

**Supplementary Information:**

The online version contains supplementary material available at 10.1186/s12889-026-27360-x.

## Background

Over the past 20 years, there has been a secular escalating tendency of childhood hypertension (HTN). Song et al. in their systematic review and meta-analysis of 47 articles to estimate the global prevalence of pediatric HTN noticed a gradual rising trend of 75%-79% between 2000 and 2015. The prevalence of childhood HTN was lowest in the 1990s (1.26%), modest in the 2000s (3.3%), and peaked in the period of 2010–2014 (6.02%) [1]. In line with Song analysis, Crouch et al. in their systematic review and meta-analysis of 41 articles from African countries among children aged 3–19 years deduced duplication of the estimated pooled prevalence of pediatric HTN after 2015 (10%) compared to before 2015 (5.6%) [2]. The global meta-analysis of childhood BP profile is alarming; it revealed an overall elevated BP and HTN prevalence of 9.67% and 4% respectively with the highest HTN prevalence in the African and Eastern Mediterranean Regions (6.94% and 5.26% respectively) [1]. The meta-analysis of BP among African children yielded an overall elevated BP and HTN prevalence of 11.38% and 7.45% respectively [2]. 

Unlike adults, the blood pressure (BP) status in children and adolescents (1–18 years) is assessed against the normative distribution of BP in healthy children after adjustment to the child sex, age, and height. Several childhood BP distribution norms have been developed. The most recent of which has been developed by the American Academy of Pediatrics (AAP) in the clinical practice guidelines for screening of the childhood BP in 2017 [3]. The BP profile in children and adolescents is staged as normal, elevated BP, stage 1, or stage 2 HTN based on readings and classification according to the percentiles. Childhood HTN is considered when the systolic BP (SBP) and/ or diastolic BP (DBP) readings are *≥* 95th percentile on at least 3 separate occasions [4]. 

Clinically, high BP in childhood is often asymptomatic and discovered incidentally. The signs and symptoms commonly associated with high BP are headache, vomiting, chest pain, palpitation, shortness of breath, and seizures [5]. Persistence of elevated BP and development of HTN in childhood dramatically damage the blood vessels of the vital organs and prematurely develop long-term complications of the cardiovascular system (left ventricular hypertrophy, coronary artery diseases and strokes), eyes, and kidneys [6, 7]. High BP in younger children is usually secondary to an underlying cause. By time, older children, like adults, are more likely to have primary HTN associated with the same unmodifiable (age, sex, family history, and ethnicity) and modifiable (excess weight, smoking, unhealthy diets, and physical inactivity) risk factors [8]. 

Obesity has been shown to be the most paramount risk factor for elevated BP during childhood. Several studies have endorsed the strong association between body mass index (BMI) and elevated BP in childhood worldwide [9, 10]. Moreover, childhood BP could be affected by different dietary habits like unhealthy high intake of saturated/trans fats, free sugars, and sodium with low consumption of fruits and vegetables [11]. In addition, strong positive correlation was revealed between sedentary behaviors and high BP levels [12]. 

Undetected high childhood BP is so common. Factors contributing to such under-recognition are unawareness of childhood high BP and its significance, work overflow, no measurement of child BP, poor staff training, complexity of the diagnostic technique, need for repeated visits for confirmation, misinterpretation of BP readings, and poor perception of primary HTN and its effects in children [13, 14]. Elevated BP in childhood is an unseen source of HTN in adulthood. BP tracking from childhood through adulthood has shown an eminent correlation between higher BP in childhood through adulthood with onset of HTN in early adulthood [15]. 

## Methods

The current study aimed to estimate the prevalence of high BP (elevated BP and HTN) among preparatory schoolchildren in El-Beheira Governorate, Egypt and to identify correlates associated with high BP.

A cross-sectional study was conducted in 4 randomly selected governmental preparatory schools in Damanhur district of El Behera Governorate, Egypt. From a previous study, the prevalence of high BP among schoolchildren was 9.7% [16]. Using margin of error of 2.5%, design effect of 1.5, and confidence level of 95%, the minimum required sample size was 807 students. The sample size was calculated using Epi-info software. The current study included apparently healthy preparatory schoolchildren aged 11–14 years at the selected schools to be eligible and could participate in the study with the exclusion of schoolchildren who had chronic debilitating diseases or did not provide their parent / legal guardian informed consent.

Sampling approach was adopted using multistage random sampling technique. At first, Damanhur was selected among the 15 districts of El-Behera governorate using simple random sampling (SRS). Then, for geographic representation, Damanhur district was subdivided geographically into four semi-equal divisions (A, B, C, & D). Using SRS, the first division was selected and titled A and assigned randomly for the selection of a girls’ preparatory school. Divisions B, C, and D were alternatively assigned to boys, girls, and boys’ preparatory schools. Then, a list of all public boys’ preparatory schools (division B and D) and girls’ preparatory schools (division A and C) was prepared for each division. From the prepared list, four preparatory schools were randomly selected (one from each prepared list): two girls’ schools from divisions A and C and two boys’ schools from divisions B and D. At each school, a list of all classes for each grade was prepared. Then, 6 classes were randomly selected (2 classes per grade) using SRS. Finally, within each class, all eligible children were included in the study. A total of 912 students were initially invited to participate in the study from the selected schools. Of them, 856 students actually participated in the current study with 93.9% response rate while the other 38 students were absent while conducting the current study or did not provide their parent / legal guardian informed consent.

All participants were interviewed for filling a pre-designed, structured questionnaire (Supplementary file 1) including the following: (a) Socio-demographic data: age, sex, class grade, parents’ level of education. (b) Personal habits of cigarettes smoking and physical activity frequency. (c) Dietary habits: adding table salt and ten questions were posted to inquire about the frequency of different food items. Except for adding table salt question, the frequency of the ten different food items [1- vegetables, 2- fruits, 3- salted food, 4- ketchup, mayonnaise, or other sauces, 5- processed meat, 6- fried food, 7- fast food, 8- chocolate and biscuits, 9- soft drinks, and 10- canned juices] was categorized as follows: never, once monthly, once weekly, 2–3 times weekly, and daily. The frequencies of never, once monthly, and once weekly were scored 1 for intake of vegetables and fruits. While the frequencies of 2–3 times weekly and daily were scored 0. Conversely the frequency of other remaining food items including were scored as 0 for never, once monthly, once weekly and 1 for 2–3 times weekly and daily. Scores of 0 and 1 indicate low risk and high risk respectively. The total score of the ten questions ranged from 0 to 10. A cutoff point of 5 was arbitrarily chosen to determine the level of dietary risk as follows: <5: low risk and *≥* 5 high risk. This was an exploratory risk score used to provide a composite indicator of overall dietary risk. The selection and development of the risk score was done by a panel of three experts in public health and nutrition based on different dietary habits that could affect childhood BP status [8, 11]. (d) Family history of chronic diseases: HTN, cardiac diseases, diabetes mellitus (DM), and obesity.

Then the following measurements were obtained: (a) Anthropometric measurements: weight in kilogram (kg), height in centimeter (cm), and waist circumference (cm) were measured according to the procedures described by Gibson 2023 [17]. BMI was calculated as weight (kg) to height in meter (m) square (kg/ m^2^). Evaluation of BMI based on the score of centile was interpreted as follows: underweight: BMI for age below 5th centile, normal: BMI for age from above or equal to 5th centile to below 85th centile, overweight: BMI for age equal to or above 85th centile but less than 95th centile, and obese: BMI for age equal to or above 95th centile [18]. Waist (cm) to height (cm) ratio was calculated. A cutoff of 0.5 was used to define abdominal obesity for both sexes [19]. (b) Auscultatory BP measurement: using a mercury sphygmomanometer (KBM, model number SM-300, Kawamoto corporation, Osaka, Japan), in the seated position with supported back, exposed right arm, with at least a 10-min rest period prior to its measurement, and using appropriate cuff bladder length (≥ 80–100%) of the child’s upper arm circumference. Three measurements were taken for each participant with 2-minute interval, and the average was recorded. Students with high BP readings (elevated BP, stage 1 HTN & stage 2 HTN) were rechecked three times on 3 consecutive days to confirm the diagnosis for those with sustained high readings over 3 visits [20]. Prior to launching the study, researchers measured BP for 20 children, who were not included in the current study, to ensure validity, agreement, quality, and standardization. BP of the studied students was classified according to the new American normative BP in healthy children and adolescents as follows: Children aged 1–13 years: Normal BP: <90th percentile, elevated BP: ≥90th - <95th percentile or 120/80 mm Hg - <95th percentile (whichever was lower), stage 1 HTN: ≥95th - <95th percentile + 12 mm Hg or 130/80–139/89 mm Hg (whichever was lower), and stage 2 HTN: ≥95th percentile + 12 mm Hg or ≥ 140/90 mm Hg (whichever was lower). Children aged > 13 years: Normal BP: <120/<80 mmHg, elevated BP: 120/<80 mmHg − 129/<80 mm Hg, stage 1 HTN: 130/ 80–139/ 89 mm Hg, and stage 2 HTN: ≥140/90 mm Hg [4]. All participants with stage 2 HTN were immediately referred for medical consultation.

### Statistical analysis

Collected data were coded, revised, cleaned, tabulated, and analyzed through IBM SPSS Statistics version 27 using the appropriate descriptive and analytical statistics. The descriptive statistics included the percentages (%), arithmetic mean (X̅), and standard deviation (SD) which described various qualitative and quantitative data of the study participants. The analytical statistics entailed the Chi squared test (χ^2^), student t test, and analysis of variance (ANOVA). All the studied variables were included in the univariate analysis while the multivariate logistic regression analysis was conducted for variables with *p* value < 0.1 to determine predictors for having high BP. Multicollinearity among all the independent variables included in the multivariate logistic regression was assessed through variance inflation factor (VIF) to ensure the validity of the regression model. All VIF values were < 2.0 (ranged from 1.024 to 1.775) indicating no evidence of multicollinearity. The statistical fitness of the multivariate logistic regression model was assessed using the Omnibus Test of Model Coefficients. The model was statistically significant, χ^2^ = 318.36, *p* < 0.05. The proportion of variance explained by the predictors was estimated using Cox & Snell and Nagelkerke R^2^ statistics. This revealed that the model explained 31.1% (Cox & Snell R^2^) to 53.1% (Nagelkerke R^2^) of the variance in BP status and correctly classified 88.9% of cases. Also, the Hosmer and Lemeshow test was applied to assess the goodness of fit, where a non-significant result (*p* > 0.05) indicated that model adequately fit the data and suggested good alignment between predictors and outcome. In all applied analytical statistical tests, *p* value ≤ 0.05 was considered significant.

## Results

A sum of 856 students equally distributed (50%) among boys and girls, of an average age of 13.3 ± 0.66 years took part in the study. Nearly half of their fathers and mothers completed secondary school education. 59%, 27%, and 14% of them practiced physical activity daily, 2–3 times/ week, and *≤* 1 time/ week, respectively. 40.4% of students reported adding table salt while eating. Based on the developed dietary risk scale, 28.9% were classified having high risk. Family history of HTN was reported by 20.1% of the students [Table 1].

**Table 1:** Socio-demographic characteristics, habits, and family history of some non-communicable diseases, of the students

Schoolchildren characteristics		Total (*n* = 856) *N* (%)
Age (years)	Min-Max	11–14
	Mean ± SD	13.3 ± 0.66
Sex	Boys	426 (49.8)
	Girls	430 (50.2)
School grade	First grade	296 (34.6)
	Second grade	279 (32.6)
	Third grade	281 (32.8)
Father’s education	Illiterate / read & write	100 (11.7)
	Primary	49 (5.7)
	Preparatory	111 (13.0)
	Secondary	449 (52.5)
	University	147 (17.1)
Mother’s education	Illiterate / read & write	124 (14.5)
	Primary	44 (5.1)
	Preparatory	70 (8.2)
	Secondary	470 (54.9)
	University	148 (17.3)
Physical activity	≤ once weekly	120 (14.0)
	2–3 times weekly	231 (27.0)
	Daily (at least 5 days/week)	505 (59.0)
Diet risk	Low risk	609 (71.1)
	High risk	247 (28.9)
Adding table salt	No	510 (59.6)
	Yes	346 (40.4)
Family history	Diabetes	137 (16.0)
	Hypertension	172 (20.1)
	Cardiac diseases	31 (3.6)
	Obesity	128 (15.0)

Table 2 demonstrated the students’ anthropometric data. These latter recorded an average of 76.78 ± 9.78 cm for waist circumference, 20.6 ± 4.42 kg/m2 BMI, and 0.48 ± 0.06 for waist to height ratio with significant higher averages among girls than boys, (*p* < 0.001). The BMI status frequency was mostly headed by normal weight (68.3%) followed by obese (13.6%) and overweight (13%). Only 34.2% of the students were diagnosed with abdominal obesity. Girls had higher frequency of Obesity (15.3% vs. 11.7%), overweight (15.8% vs. 10.1%) and abdominal obesity (40.5% vs. 27.9%) than boys, (*p* < 0.001).

**Table 2:** Students’ anthropometric data

Anthropometric data		Total (*n* = 856)	Boys (*n* = 426)	Girls (*n* = 430)	Statistical Test (*p*-value)
		*N* (%)	*N* (%)	*N* (%)	
Waist circumference	Min-Max	49–112	58–112	49–108	t = -3.944 (< 0.001)**
	Mean ± SD	76.78 ± 9.78	75.47 ± 9.46	78.08 ± 9.92	
Body mass index (BMI)	Min-Max	12.7–38.8	12.7–38.2	13.7–38.8	t = -6.034 (< 0.001)**
	Mean ± SD	20.6 ± 4.42	19.7 ± 3.97	21.49 ± 4.66	
BMI status	Underweight	44 (5.1)	33 (7.7)	11 (2.6)	χ^2^ = 19.204 (< 0.001)**
	Normal	585 (68.3)	300 (70.4)	285 (66.3)	
	Overweight	111 (13.0)	43 (10.1)	68 (15.8)	
	Obese	116 (13.6)	50 (11.7)	66 (15.3)	
Waist to height ratio	Min-Max	0.29–0.68	0.36–0.68	0.29–0.68	t = -4.589 (< 0.001)**
	Mean ± SD	0.48 ± 0.06	0.47 ± 0.05	0.49 ± 0.06	
Abdominal obesity	No	563 (65.8)	307 (72.1)	256 (59.5)	χ^2^ = 14.926 (< 0.001)**
	Yes	293 (34.2)	119 (27.9)	174 (40.5)	

** *p* ≤ 0.01 is highly statistically significant

**Fig. 1:**
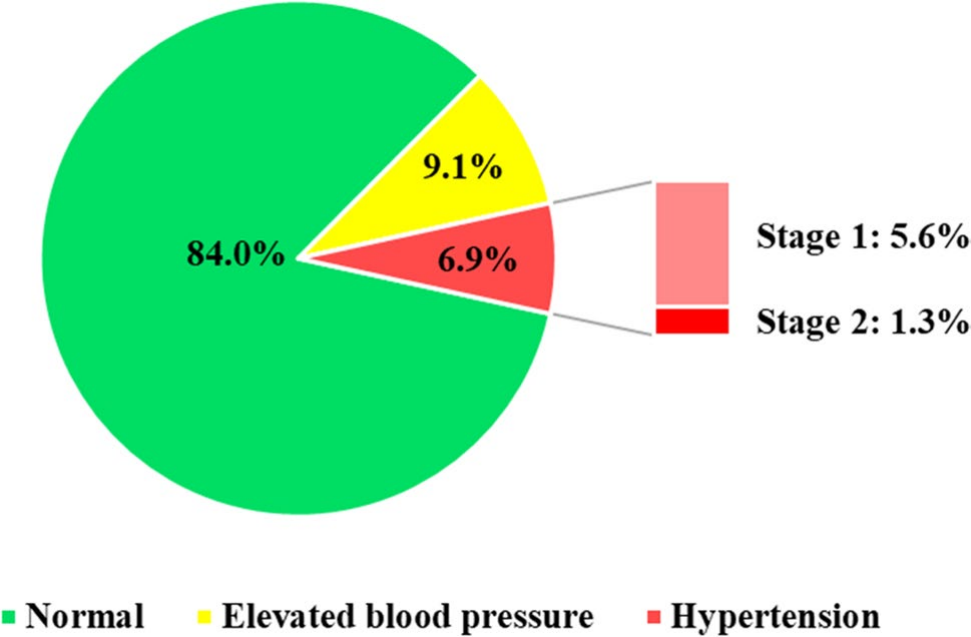
Students’ blood pressure status after 3 measures for diagnosis

Table 3; Fig. 1 displayed the students’ BP status according to the new American normative BP in healthy children and adolescents. At the first reading, 78.4%, 4.4%, and 17.2% of students were respectively classified to have normal BP, elevated BP and HTN. At the third reading, the corresponding values changed to 84%, 9.1%, and 6.9% respectively.

**Table 3:** Students’ blood pressure status

Blood pressure status	First reading		Second reading		Diagnosis after 3 readings	
	*N*	%	*N*	%	*N*	%
Normal	671	78.4	692	80.8	719	84.0
Elevated blood pressure	38	4.4	63	7.4	78	9.1
Hypertension	147	17.2	101	11.8	59	6.9
• Stage 1 hypertension	105	12.3	73	8.5	48	5.6
• Stage 2 hypertension	42	4.9	28	3.3	11	1.3

In Table 4, among the socio-demographic characteristics, only age and sex owed significant relationship to BP. The average age of the students studied was 13.27 ± 0.66 years for normal BP group, 13.49 ± 0.62 elevated BP group, and 13.46 ± 0.6 for hypertensive group with overall significant difference, *p* = 0.004. Moreover, salient higher frequency of HTN (10%) and elevated BP (12.6%) among girls in comparison to boys (3.8% & 5.6%) was evident, *p* < 0.001. There was a significant higher frequency of HTN and elevated BP among students practicing physical activity *≤*once a week (17.5% & 25%) than those practicing physical activity 2–3 times a week (14.7% & 17.3%) and daily (0.8% & 1.6%), (*p* < 0.001). Further, there was a significant higher frequency of HTN and elevated BP among students classified as having high-risk diet (15% & 16.6%) and those used added table salt (15.6% & 18.5%) compared to those classified as having low risk diet (3.6% & 6.1%) and those did not use added table salt (1% & 2.7%), *p* < 0.001. The recall revealed that the only significant linkage of positive HTN family history was to each of HTN (HTN: 12.8% vs. 5.4%) and elevated BP (11.6% vs. 8.5); χ^2^ = 14.172 (*p* < 0.001). Waist circumference, BMI, and waist to height ratio were the highest among students with HTN (87.66 ± 11.5 cm, 25.92 ± 5.72 kg/m^2^, & 0.54 ± 0.07), followed by those with elevated BP (84.32 ± 10.64 cm, 24.14 ± 4.84 kg/m^2^, & 0.52 ± 0.06), and lowest with those with normal BP (75.07 ± 8.5 cm, 19.78 ± 3.7 kg/m^2^, & 0.47 ± 0.05) and the differences in-between was statistically significant, *p* < 0.001. In exact terms, the frequency of HTN and elevated BP was significantly higher among obese children (24.1% & 21.6%) and overweight children (9.9% & 21.6%) compared to normal weight ones (3.4% & 5.6%), *p* < 0.001. Furthermore, the frequency of HTN and elevated BP was significantly higher among children with abdominal obesity (13.7% & 15.4%) compared to those without (3.4% & 5.9%), *p* < 0.001.

**Table 4:** Relationship of students’ blood pressure status to socio-demographic characteristics, habits, family history, and anthropometric measurements

Schoolchildren characteristics		Blood pressure status *N* (%)			Statistical Test (*p*-value)
	Total (*n* = 856)	Normal	Elevated BP	Hypertension	
Age (years)	Min-Max	11–14	12–14	12–14	F = 5.548 (0.004)**
	Mean ± SD	13.27 ± 0.66	13.49 ± 0.62	13.46 ± 0.6	
Sex	Boys (*n* = 426)	386 (90.6)	24 (5.6)	16 (3.8)	χ^2^ = 27.783 (< 0.001)**
	Girls (*n* = 430)	333 (77.4)	54 (12.6)	43 (10.0)	
School grade	First grade (*n* = 296)	259 (87.5)	23 (7.8)	14 (4.7)	χ^2^ = 6.642 (0.156)
	Second grade (*n* = 279)	235 (84.2)	23 (8.3)	21 (7.5)	
	Third grade (*n* = 281)	225 (80.1)	32 (11.4)	24 (8.5)	
Father’s education	Illiterate / read & write (*n* = 100)	86 (86.0)	8 (8.0)	6 (6.0)	χ^2^ = 4.53 (0.806)
	Primary (*n* = 49)	43 (87.7)	4 (8.2)	2 (4.1)	
	Preparatory (*n* = 111)	89 (80.2)	14 (12.6)	8 (7.2)	
	Secondary (*n* = 449)	381 (84.9)	39 (8.7)	29 (6.4)	
	University (*n* = 147)	120 (81.6)	13 (8.9)	14 (9.5)	
Mother’s education	Illiterate / read & write (*n* = 124)	102 (82.3)	12 (9.6)	10 (8.1)	χ^2^ = 3.717 (0.882)
	Primary (*n* = 44)	39 (88.6)	2 (4.6)	3 (6.8)	
	Preparatory (*n* = 70)	59 (84.3)	8 (11.4)	3 (4.3)	
	Secondary (*n* = 470)	396 (84.3)	44 (9.3)	30 (6.4)	
	University (*n* = 148)	123 (83.1)	12 (8.1)	13 (8.8)	
Physical activity	≤ once weekly (*n* = 120)	69 (57.5)	30 (25.0)	21 (17.5)	χ^2^ = 177.358 (< 0.001)**
	2–3 times weekly (*n* = 231)	157 (68.0)	40 (17.3)	37 (14.7)	
	Daily (at least 5 days/week) (*n* = 505)	493 (97.6)	8 (1.6)	4 (0.8)	
Diet risk	Low risk (*n* = 609)	550 (90.3)	37 (6.1)	22 (3.6)	χ^2^ = 64.327 (< 0.001)**
	High risk (*n* = 247)	169 (68.4)	41 (16.6)	37 (15.0)	
Adding table salt	No (*n* = 510)	491 (96.3)	14 (2.7)	5 (1.0)	χ^2^ = 142.768 (< 0.001)**
	Yes (*n* = 346)	228 (65.9)	64 (18.5)	54 (15.6)	
Diabetes family history	No (*n* = 719)	602 (83.7)	67 (9.3)	50 (7.0)	χ^2^ = 0.273 (0.872)
	Yes (*n* = 137)	117 (85.4)	11 (8.0)	9 (6.6)	
Hypertension family history	No (*n* = 684)	589 (86.1)	58 (8.5)	37 (5.4)	χ^2^ = 14.172 (< 0.001)**
	Yes (*n* = 172)	130 (75.6)	20 (11.6)	22 (12.8)	
Cardiac diseases family history	No (*n* = 825)	693 (84.0)	76 (9.2)	56 (6.8)	χ^2^ = 0.612 (0.736)
	Yes (*n* = 31)	26 (83.8)	2 (6.5)	3 (9.7)	
Obesity family history	No (*n* = 728)	613 (84.2)	66 (9.1)	49 (6.7)	χ^2^ = 0.221 (0.895)
	Yes (*n* = 128)	106 (82.8)	12 (9.4)	10 (7.8)	
Waist circumference	Min-Max	49–105	59–112	60–108	F = 84.501 (< 0.001)**
	Mean ± SD	75.07 ± 8.5	84.32 ± 10.64	87.66 ± 11.5	
Body mass index (BMI)	Min-Max	12.7–36.1	14.9–37.4	16.7–38.8	F = 98.687 (< 0.001)**
	Mean ± SD	19.78 ± 3.7	24.14 ± 4.84	25.92 ± 5.72	
BMI status	Underweight (*n* = 44)	43 (97.7)	1 (2.3)	0 (0.0)	χ^2^ = 120.414 (< 0.001)**
	Normal (*n* = 585)	532 (91)	33 (5.6)	20 (3.4)	
	Overweight (*n* = 111)	81 (73.0)	19 (17.1)	11 (9.9)	
	Obese (*n* = 116)	63 (54.3)	25 (21.6)	28 (24.1)	
Waist to height ratio	Min-Max	0.29–0.65	0.37–0.68	0.37–0.68	F = 52.393 (< 0.001)**
	Mean ± SD	0.47 ± 0.05	0.52 ± 0.06	0.54 ± 0.07	
Abdominal obesity	No (*n* = 563)	511 (90.7)	33 (5.9)	19 (3.4)	χ^2^ = 57.575 (< 0.001)**
	Yes (*n* = 293)	208 (70.9)	45 (15.4)	40 (13.7)	

** *p* ≤ 0.01 is highly statistically significant

Univariate analysis of variables showed prominent association between high BP and age (crude odds ratio (COR) = 1.651, 95% confidence interval (CI); 1.233–2.229), girls (COR = 2.811, 95% CI; 1.891–4.178), third school grade (COR = 1.742, 95% CI; 1.109–2.738), physically activity ≤ once weekly (COR = 30.366, 95% CI; 15.424–59.785) & 2–3 times weekly physical activity (COR = 19.364, 95% CI; 10.253–36.571), high risk diet (COR = 4.302, 95% CI; 2.944–6.289), usage of added table salt (COR = 13.374, 95% CI; 8.037–22.257), positive family history of HTN (COR = 2.003, 95% CI; 1.330–3.018), and being overweight (COR = 3.718, 95% CI; 2.243–6.161) and obese (COR = 8.444, 95% CI; 5.322–13.399), and abdominal obesity (COR = 4.016, 95% CI; 2.744–5.877), (p < 0.05). While the multivariate analysis including the abovementioned variables revealed that physically activity ≤ once weekly (adjusted odds ratio (AOR) = 15.679, 95% CI; 6.936–35.443) & 2–3 times weekly physical activity (AOR = 10.738, 95% CI; 5.247–21.973), usage of added table salt (AOR = 5.745, 95% CI; 3.108–10.617), being overweight (AOR = 2.735, 95% CI; 1.298–5.765) and obese (AOR = 7.463, 95% CI; 3.414–16.314) were the predictors for having high BP level among the students, (*p *< 0.05) [Table 5].

**Table 5:** Univariate and multivariate logistic regression analysis of students’ high BP predictors

Total (*n* = 856)		Crude OR (95% CI; LL-UL)	*p* value	Adjusted OR (95% CI; LL-UL)	*p* value
Age		1.651 (1.223–2.229)	0.001**	1.690 (0.971–2.943)	0.064
Sex	Boys (*n* = 426)	Reference			
	Girls (*n* = 430)	2.811 (1.891–4.178)	< 0.001**	0.925 (0.516–1.659)	0.794
School Grade	First (*n* = 296)	Reference			
	Second (*n* = 279)	1.311 (0.818-2.100)	0.261	1.433 (0.669–3.070)	0.354
	Third (*n* = 281)	1.742 (1.109–2.738)	0.016*	1.181 (0.534–2.612)	0.681
Father’s Education	Illiterate/read&write (*n* = 100)	Reference			
	Primary (*n* = 49)	0.857 (0.308–2.387)	0.768		
	Preparatory (*n* = 111)	1.518 (0.730–3.159)	0.264		
	Secondary (*n* = 449)	1.096 (0.589–2.040)	0.772		
	University (*n* = 147)	1.382 (0.685–2.790)	0.366		
Mother’s Education	Illiterate/read&write (*n* = 124)	Reference			
	Primary (*n* = 44)	0.594 (0.210–1.680)	0.326		
	Preparatory (*n* = 70)	0.864 (0.392–1.908)	0.718		
	Secondary (*n* = 470)	0.866 (0.513–1.462)	0.591		
	University (*n* = 148)	0.942 (0.502–1.770)	0.853		
Physical Activity	≤ once weekly (*n* = 120)	30.366 (15.424–59.785)	< 0.001**	15.679 (6.936–35.443)	< 0.001**
	2–3 times weekly (*n* = 231)	19.364 (10.253–36.571)	< 0.001**	10.738 (5.247–21.973)	< 0.001**
	Daily (*n* = 505)	Reference			
High diet risk (*n* = 247)		4.302 (2.944–6.289)	< 0.001**	1.408 (0.845–2.344)	0.189
Adding table salt (*n* = 346)		13.374 (8.037–22.257)	< 0.001**	5.745 (3.108–10.617)	< 0.001**
Positive family history of DM (*n* = 137)		0.880 (0.526–1.470)	0.624		
Positive family history of HTN (*n* = 172)		2.003 (1.330–3.018)	< 0.001**	1.451 (0.834–2.525)	0.188
Positive family history of cardiac diseases (*n* = 31)		1.010 (0.381–2.677)	0.985		
Positive family history of obesity (*n* = 128)		1.106 (0.671–1.825)	0.692		
BMI status	Underweight (*n* = 44)	0.233 (0.032–1.729)	0.154	0.683 (0.076–5.420)	0.683
	Normal (*n* = 585)	Reference			
	Overweight (*n* = 111)	3.718 (2.243–6.161)	< 0.001**	2.735 (1.298–5.765)	0.008**
	Obese (*n* = 116)	8.444 (5.322–13.399)	< 0.001**	7.463 (3.414–16.314)	< 0.001**
Abdominal obesity (*n* = 293)		4.016 (2.744–5.877)	< 0.001**	1.221 (0.631–2.360)	0.554

*OR* Odds ratio, *CI* Confidence interval, *LL* Lower limit UL: Upper limit

* *p* ≤ 0.05 is statistically significant

** *p* ≤ 0.01 is highly statistically significant

## Discussion

The analysis of Song et al. revealed an overall 4% global pooled prevalence of pediatric HTN with higher prevalence in low- and middle-income countries (LMICs) compared to high-income countries (HIC). Also, the highest HTN prevalence was noticed in the African (6.94%) and East Mediterranean Regions (5.26%) [1]. The pooled prevalence of pediatric HTN among African children was almost doubled (7.45%) according to Crouch et al. [2]

Country wise, the prevalence of pediatric HTN was quite variable. It was almost 6% in Iran, China, and India [21–23]. Slightly higher prevalence of pediatric HTN (8%) was reported in Egypt and India [16, 24]. Further increased prevalence of pediatric HTN was noticed in Tanzania (10.8%), Jazan, Saudi Arabia (11.6%), and in India (12.4%) [25–27]. A much higher prevalence of pediatric HTN was recorded in Turkey (14.8%), Jeddah University Hospital, Saudi Arabia (15.2%), and in Tunisia (15.4%) [28–30]. An Extremely higher prevalence of pediatric HTN was reported in India (19.7% and 23%) [31, 32]. On the contrary, low prevalence of pediatric HTN was noted in cross-sectional studies in Cameron (1.6%) and in India (2.7%) [33, 34]. In line with Ebrahimi et al. [21], Lu et al. [22], and Patel et al. [23], the current study yielded a prevalence of childhood HTN of 6.9% among the studied students.

Similar to pediatric HTN, elevated pediatric BP prevalence varied country wise. It was lowest in India (3.2%) [34] and Tanzania (4.4%) [25], modest in Jeddah, Saudi Arabia (6%) [29], China (6.6%) [22], India (6.9%) [23], Iran (7.4%) [21], and in Cameron (8.1%) [33], and highest in Turkey (11.2%) [28], Jazan, Saudi Arabia (12%) [26], , Tunisia (12.4%) [30], India (13.4%) [31], and in Egypt (14.7%) [16]. An extremely higher prevalence of elevated pediatric BP (21.6%) was seen in India [24]. In line with Song et al. [1], Crouch et al. [2], Ebrahimi et al. [21], Chelo et al. [33], Çam and Ustuner [28], El-Setouhy et al. [26], , and Soua et al. [30], the current study revealed a prevalence of elevated pediatric BP of 9.1% among the studied students.

The discrepancy in the prevalence of pediatric HTN and elevated BP could be explained on the basis of variation in the research approach, procedures, and confounders. The influence of adherence to the standardized three separate readings technique of BP measurement for the diagnosis of pediatric HTN was evident. As it helps a lot to achieve accurate diagnosis of pediatric HTN, avoid white coat HTN, and avert other conditions such as child activity and emotional state and the surrounding circumstances that might interfere with BP measurement [4]. 

Although BP was measured on three consecutive days, the prevalence of HTN dropped by approximately 60% from 17.2% on the first reading to 11.8% on the second reading and reached 6.9% on the third reading. The significance of adherence to the three readings of childhood BP to diagnose HTN was quite evident in Sun et al. systematic review and meta-analysis of 21 studies including 179,561 children [35]. Similar findings were endorsed by Marcovecchio et al. and Zhang et al. in their cross-sectional studies [36, 37]. Deviation from the standardized 3-reading protocol for BP measurement could be the main contributor to the extreme high prevalence of pediatric HTN in the studies of Das et al. [31] who considered HTN based on the mean of 3 BP readings in the same visit and Narang et al. [32] who diagnosed HTN based on only 2 readings in the same visit separated by 5-minute interval.

The role of other confounding factors shared the responsibility for the discrepancy of pediatric HTN prevalence. The influence of age of the study participants was well obvious. Inclusion of younger children diluted the prevalence of pediatric HTN [33, 34], while the inclusion of older children cumulated it [27–30]. The impact of the study settings on childhood HTN prevalence was evident by Ghamri et al. who conducted their study at health facility in Jaddah [29]. 

Age is a major contributor to development and progression of HTN. Compared to younger children, older ones had higher HTN and elevated BP prevalence [16]. Again, age correlated positively with both SBP and DBP among children [29]. Also, children with high BP possessed statistically significantly higher average age than normal BP [22, 25]. In congruence with the previous studies, the current study showed higher average age among elevated BP children and hypertensive children compared to normotensive ones.

The impact of sex on childhood HTN and elevated BP was obvious. Prominent higher frequency of HTN and elevated BP in girls in comparison to boys was evident in the current study. Salient prevalence of elevated BP and HTN among girls in comparison to boys was well evident in Crouch et al. meta-analysis [2], and cross-sectional studies in Saudi Arabia [26, 29], India [27, 31, 32], and in Iran [21]. Conversely, higher prevalences of pediatric HTN and elevated BP were documented among boys than girls in Egypt and Tunisia [16, 30]. The higher frequency of childhood HTN among girls than boys logically coincided with the higher frequency of adulthood HTN among women than men in Egypt as mentioned in the World Health Organization (WHO) 2023 Report and Fares and Soliman cross-sectional study [38, 39]. Besides, it was evident in the current study that girls had significantly higher frequency of obesity, overweight and abdominal obesity than boys.

Hypertension often occurs in familial clusters and seems to be heritable. Remarkable inclination of both childhood HTN and elevated BP towards positive family than the negative one was well seen in the current study. Concomitant to the current results, solid connection between childhood HTN and elevated BP and positive family history was noted in Egypt [16], Saudi Arabia [26, 29], and in India [27]. 

Physical inactivity is an eminent modifiable risk factor for HTN. Compared to physically active children, inactive ones showed higher prevalence of HTN and elevated BP in Egypt and China [16, 22]. Concomitantly, the current study ascertained this relationship. The contribution of dietary habits to development of childhood HTN has been widely studied worldwide. A significant observational connection between consumption of foods poor in vegetables and fruits and rich in fried salty proteins to HTN was exhibited [24, 40]. In the same context, the current study found significantly higher frequency of HTN and elevated BP among students classified as high-risk diet and those used added table salt compared to those classified as low risk diet and those did not use added table salt.

Worldwide, the sacred union between obesity / overweight and high BP readings has been strongly affirmed. Higher prevalence of elevated BP and HTN among obese / overweight children than normal weight ones was inferred in Africa [2], Egypt [16], Saudi Arabia [29], Tunisia [30], India [24, 31], and in Tanzania [25]. Likewise, the frequency of elevated BP and HTN was highest, modest, and lowest among obese, overweight, and normal weight children respectively in different studies [1, 26, 28, 32, 40]. Additionally, higher prevalence of elevated BP and HTN was noted among children with abdominal obesity than non-obese ones [16, 28]. The SBP and DBP readings positively correlated with each of waist circumference and waist to height ratio in India [32]. Moreover, hypertensive Chinese children owed higher average waist circumference and waist to height ratio in comparison to those with normal BP [22, 40]. In congruence, the current study affirmed this prominent association.

The multivariate analysis accredited the predictivity of different correlates worldwide. It attested the predictivity of high waist circumference, overweight and obesity, family history of HTN, lack of regular physical activity, and increasing age were the significant predictors for childhood HTN in Egypt; [16] obesity and overweight in Saudi Arabia [26], and overweight/ obesity and family history of HTN in Saudi Arabi [29], obesity and overweight in Turkey [28], and overweight/obesity and high consumption of salted food in India [24]. In concordance with the previous studies, the multivariate analysis in the current study confirmed the predictivity of physically activity ≤ once weekly & 2–3 times weekly physical activity, usage of added table salt, being overweight and obese for having high BP level among the students. The consistency of obesity and overweight predictivity in the current and previous studies could be ascribed to the dependence on the actual objective anthropometric measurements (weight, height, and waist circumference) unlike the subjective family history, physical activity, and dietary habits data that relied on the recall.

The study had a limitation of cross-sectional design adoption which cannot establish a definitive causal link between risk factors and high BP; however, this is strongly mitigated by the solid and rigorous methodology employed. Despite following the AAP 2017 guidelines for BP status classification, BP measurements were done over three consecutive days for confirmation rather than the application of measurement over three separate occasions. Additionally, dietary intake and physical activity were self-reported that might introduce measurement limitation because of the recall bias. However, data were collected through interviewing questionnaire instead of the self-administered questionnaire to ensure response and quality of the data. The current study also ascertained the association between HTN and studied variables that might need further longitudinal follow up studies to establish this causality. Moreover, the study restricted to only one Egyptian Governorate. To be generalizable to the overall Egyptian situation, the study needs to be replicated to represent all Egyptian Governorates. However, this is offset by the large and representative sample size of 856 students. The use of a multi-stage random sampling technique and a sample size that exceeded the statistical requirement ensured that the data is highly reliable and provided a precise reflection of the pediatric population, offering a strong foundation for future national-scale research.

## Conclusions

Screening of schoolchildren has explicitly unveiled the high frequency of high BP of elevated BP and HTN among preparatory schoolchildren. Strikingly, age, female sex, family history of HTN, physical inactivity, bad dietary habits, overweight, obesity, and abdominal obesity correlated robustly with childhood elevated BP and HTN. These associations emphasized the importance of lifestyle modification in rectifying childhood high BP levels. Pursuant to the current study, it is critical to apply preventive strategies that emphasize awareness, screening, and early detection of childhood elevated BP and HTN. This can be achieved through the conduction of a nationwide multicenter cross-sectional study for early detection of childhood elevated BP and HTN and development of an Egyptian childhood normative BP profile. Besides, further longitudinal prospective cohort studies are needed to track children over time to establish causality of the identified associated factors of high BP.

## Supplementary Information

Supplementary Material 1.

## Data Availability

Data is available on reasonable request from the corresponding author.

## Abbreviations

AAP: American Academy of Pediatrics
ANOVA: Analysis of Variance
AOR: Adjusted Odds Ratio
BMI: Body Mass Index
BP: Blood Pressure
CI: Confidence Interval
cm: Centimeter
COR: Crude Odds Ratio
DBP: Diastolic Blood Pressure
DM: Diabetes mellitus
HTN: Hypertension
kg: Kilogram
LMIC: Low- and Middle-Income Countries
m: Meter
OR: Odds ratio
SBP: Systolic Blood Pressure
SD: Standard deviation
SRS: Simple Random Sampling
VIF: Variance Inflation Factor
WHO: World Health Organization
X̅: Arithmetic mean
χ^2^: Chi squared test
