# Supplementary material for: Prevalence and predictors of pediatric high blood pressure: an Egyptian school-based survey
Source: BMC Public Health. 2026 Apr 23;26:1357. doi: 10.1186/s12889-026-27360-x (PMC13107762; doi:10.1186/s12889-026-27360-x)
Supplement: Supplementary file 1 — Supplementary Material 1. [file 12889_2026_27360_MOESM1_ESM.docx]

| **Questionnaire** | | | | | |
| --- | --- | --- | --- | --- | --- |
| **(a) Socio-demographic characteristics** | | | | | |
| **Age**: ………. (in years) | | | | | |
| **Sex**: (1) Boys (2) Girls | | | | | |
| **School grade**: (1) First grade (2) Second grade (3) Third grade | | | | | |
| **Father’s level of education**: (1) Illiterate/read & write (2) Primary (3) Preparatory (4) Secondary (5) University | | | | | |
| **Mother’s level of education**: (1) Illiterate/read & write (2) Primary (3) Preparatory (4) Secondary (5) University | | | | | |
|  | | | | | |
| **(b) Personal habits** | | | | | |
| **Cigarettes smoking**: (1) No (2) Yes | | | | | |
| **Physical activity frequency**: (1) ≤ once weekly (2) 2-3 times weekly (3) Daily (at least 5 days/week) | | | | | |
|  | | | | | |
| **(c) Dietary habits** | | | | | |
| **Adding table salt**: (1) No (2) Yes | | | | | |
|  | | | | | |
| **Frequency of eating**: | **(1) Never** | **(2) Once monthly** | **(3) Once weekly** | **(4) 2-3 times weekly** | **(5) Daily** |
| (1) Vegetables |  |  |  |  |  |
| (2) Fruits |  |  |  |  |  |
| (3) Salted food |  |  |  |  |  |
| (4) Ketchup, mayonnaise, or other sauces |  |  |  |  |  |
| (5) Processed meat |  |  |  |  |  |
| (6) Fried food |  |  |  |  |  |
| (7) Fast food |  |  |  |  |  |
| (8) Chocolate and biscuits |  |  |  |  |  |
| (9) Soft drinks |  |  |  |  |  |
| (10) Canned juices |  |  |  |  |  |
|  |  |  |  |  |  |
| **(d) Family history of:** (1) Hypertension (2) Cardiac disease (3) Diabetes mellites (4) Obesity | | | | | |
